# Blockchain-secure patient Digital Twin in healthcare using smart contracts

**DOI:** 10.1371/journal.pone.0286120

**Published:** 2024-02-29

**Authors:** Sandro Amofa, Qi Xia, Hu Xia, Isaac Amankona Obiri, Bonsu Adjei-Arthur, Jingcong Yang, Jianbin Gao

**Affiliations:** School of Computer Science and Engineering, University of Electronic Science and Technology of China, Chengdu, China; Al-Balqa Applied University Prince Abdullah bin Ghazi Faculty of Information Technology, JORDAN

## Abstract

Modern healthcare has a sharp focus on data aggregation and processing technologies. Consequently, from a data perspective, a patient may be regarded as a timestamped list of medical conditions and their corresponding corrective interventions. Technologies to securely aggregate and access data for individual patients in the quest for precision medicine have led to the adoption of Digital Twins in healthcare. Digital Twins are used in manufacturing and engineering to produce digital models of physical objects that capture the essence of device operation to enable and drive optimization. Thus, a patient’s Digital Twin can significantly improve health data sharing. However, creating the Digital Twin from multiple data sources, such as the patient’s electronic medical records (EMR) and personal health records (PHR) from wearable devices, presents some risks to the security of the model and the patient. The constituent data for the Digital Twin should be accessible only with permission from relevant entities and thus requires authentication, privacy, and provable provenance. This paper proposes a blockchain-secure patient Digital Twin that relies on smart contracts to automate the updating and communication processes that maintain the Digital Twin. The smart contracts govern the response the Digital Twin provides when queried, based on policies created for each patient. We highlight four research points: access control, interaction, privacy, and security of the Digital Twin and we evaluate the Digital Twin in terms of latency in the network, smart contract execution times, and data storage costs.

## 1 Introduction

A Digital Twin is a data-driven model of a physical asset, process, or system [1] with a persistent data connection between the physical object and model. This enables sensory data from the physical object to create increasingly detailed virtual models that reveal detective, preventative, or corrective insights for optimized operations [2]. There are several benefits to Digital Twins and the barrier to their creation is getting lower by the day due to the availability of digital tools for data collection and analytics, increasing computing power, and diminishing costs of cloud data storage.

**Fig 1:**
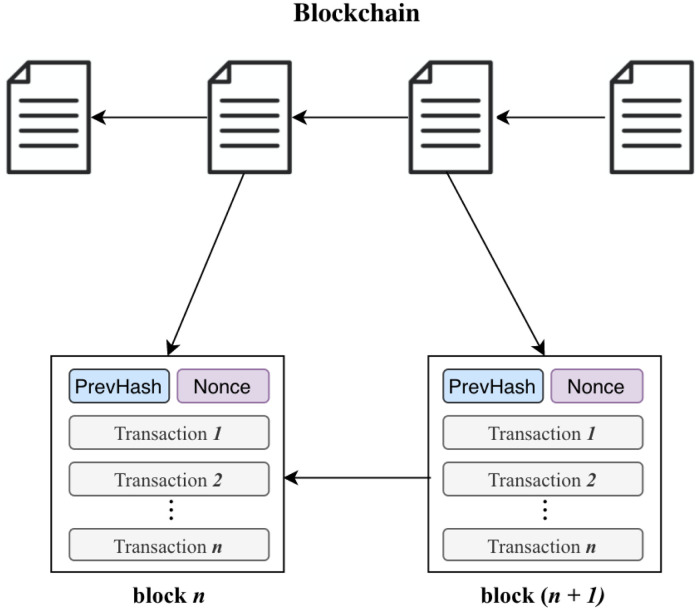
Digital Twin instances from multi-data sources.

Digital Twins are used in manufacturing for quality control and optimization purposes [3] where they permit virtual objects to be created and tested by subjecting them to exploitative, destructive experiments which may not be permitted in the physical world due to prohibitive financial costs, ethical or legal implications, etc. [4]. Thus, the application of Digital Twin technology to healthcare can bring many benefits, as experiments can be performed on data models to determine optimal treatments before prescribing them to the patient. Using available patient electronic medical records, data from smart devices, computing power, and analytics algorithms, a Digital Twin can be created for the patient, as shown in Fig 1. With more data, better approximations of the patient Digital Twin can be produced and queried to provide better access to healthcare at reduced costs and enable a higher quality of life for patients.

However, Digital Twins raise difficult questions regarding security and privacy [5]. The data connection between the patient and the Digital Twin creates the computational model of the patient’s condition(s). Inevitably, without adequate security, inaccurate data input to the Digital Twin can have serious consequences for the patient since his/her treatment may be based on the output the Digital Twin demonstrates. Additionally, unauthorized access to the Digital Twin can reveal compromising details about the patient that may cause long-term damage to their reputation with adverse financial consequences. Hence, it is critical to demand guarantees on the security of the Digital Twin and also to restrict the information it provides when queried. This research proposes a mechanism to handle four key research points: access control, interaction, privacy, and security of the Digital Twin. The need for secure data, reliable storage, trusted computing and other cooperative mechanisms with mutually beneficial interactions can be addressed using the blockchain [6–9] where a network of institutions and users collaborate to share data, collectively confer consensus-based validity on transactions, and maintain a single coherent transactions history. Provenance of data and transactions can also be guaranteed since the blockchain provides rigorous mechanisms for data integrity using digital signatures and cryptographic hashes with timestamps [10]. Periodic updates are critical for Digital Twins, thus it is important to set limits on the data the Digital Twin can receive or provide when queried. Hence, we propose a blockchain-based Digital Twin that can model the individual patient’s condition(s) and facilitate care by accessing specific services. We employ a suite of smart contracts to mediate access to the data stores from which the Digital Twin is updated.

### 1.1 Contribution

We outline our main contribution to the use of Digital Twins in healthcare below:

1. We present an automated, blockchain-based patient Digital Twin that uses smart contracts to mediate access to the Digital Twin and control its interactions.
2. We present a mathematical model of the patient Digital Twin defining it with a focus on timestamped instances.
3. We present a novel Multi-receiver Identity-Based Signcryption (mIBSC) scheme to secure the patient Digital Twin which has a constant ciphertext size and an element that can be stored on the blockchain to prove the authorship of digital twin.

The remainder of the paper is organized as follows: Section 2 presents Related Works while Section 3 provides Preliminaries dealing with the Blockchain, Digital Twins and cryptographic assumptions. Section 4 deals with the System Overview and component interactions. Section 5 discusses System Design and Section 6 presents Security proofs for our research. Section 7 provides the Efficiency Evaluation of the computations implemented and some metrics. Section 8 concludes the research and offers directions for future works.

## 2 Related works

In this section we review Digital Twins usage in healthcare with a focus on secure patient data sharing using smart contracts. In [11], the authors continuously monitor patients’ conditions and improve patient outcomes, quality of life and reduce financial costs by using Digital Twin technology for healthcare. According to this research, health monitoring can be achieved using wearable sensors for early detection of worsening health or manage chronic conditions. Furthermore, assistive technologies and increasing usage of real-time data can enable new and dynamic health services with minimal risk for the patient. The research also proposes fast simulations of conditions using machine learning for accurate crisis prediction. Thus, doctors can use the patient’s Digital Twin as a planning tool for intelligent control and emergency response. In [12], the authors define questions surrounding the materialisation, expectation and the implementation of Digital Twins in healthcare. They conclude Digital Twins can provide a useful test platform for enabling preventive healthcare for patients through simulations that employ trial-and-error so that consequences of given treatments can be evaluated empirically before the actual treatment is administered to patients. This provides a cost-effective implementation of precision medicine that offers patients the possibility of personalized treatments. For [13], the researchers tackle the integration of Digital Twins with agents and multi-agent systems. They focused on the design of agent-based Digital Twins and their utility in the context of healthcare management. They highlight the importance of Digital Twins using a case study where contextual Digital Twins of a trauma victim alert emergency medical staff in a hospital with vital details about the incoming patient. The authors of A Blockchain-based Secure Digital Twin Framework for Smart Healthy City propose a Digital Twin framework that consists of three layers: a Device Layer, a Blockchain Layer and an Application Layer. They discuss the application of their framework to the COVID-19 pandemic and highlight its suitability for use with other future public health emergencies. It provided a sequence diagram that shows how two Digital Twins and a hospital could collaborate to exchange public keys necessary for notification in case of confirmed infection. However, they do not provide proof for encryption and other security-related algorithms necessary to protect a Digital Twin [14]. This article [15] is concerned with the lack of a data collection mechanism that adequately addresses the challenge of fusing data from multiple disparate sources. The research then describes a concrete computational model of a Digital Twin for healthcare, proposes a Healthcare Digital Twin (HDT) system, and defines the protocol progression for the framework that corresponds to the mathematical model. Being a conceptual model, it provides no experimental results for any of the interactions of the Digital Twins.

We considered [16–19] for their insights on coupling Digital Twins with blockchain technology. While we appreciate their views, they do not apply to healthcare or patients. The literature agrees that the security of the Digital Twin is essential. Still, there are few proposals on guaranteeing the patient Digital Twin’s privacy and security. Thus, our research uses smart contracts to mediate access to and control of the patient’s Digital Twin.

### 2.1 Broadcast encryption

Fiat and Naor first introduced the concept of broadcast encryption (BE) or multi-receivers encryption in [20]. They proposed a method for securely encrypting a message for a group of users so that only those in the group could decrypt it. On the other hand, a coalition of non-set users cannot obtain any information about the broadcast message. Many BE methods have been developed in the contexts of identity-based encryption [21–23] and standard public-key encryption [24]. Delerablée [21] introduced the first identity-based broadcast encryption (IBBE) system with constant-size ciphertexts and private keys in the identity-based encryption context. The approach, however, does not provide ciphertext authentication. The properties of authentication and secrecy are both necessary for sharing sensitive data, such as electronic medical data. BE with source authentication is also called authenticated BE or broadcast signcryption. Selvi et al. [25] proposed an efficient identity-based signcryption scheme in this area. Similar to broadcast signcryption, there have been several multi-receiver signcryption schemes [26, 27], where the ciphertexts are of a size linear in the number of the set of receivers. Broadcast authentication schemes have also been proposed without supporting the confidentiality of the broadcast message in the public key setting [23]. Recently, Yang et al. [28] proposed a multi-message and multi-receiver signcryption scheme based on blockchain. The scheme enables medical data providers to send messages to multiple data requesters by executing one signcryption operation, which satisfies the multi-message sending requirements of the data providers in the communication environment. However, the ciphertexts are linear in the number of sets of receivers. Note that our case requires that the data owner use the blockchain to track the sequence of the patient’s Digital Twin data so that each care provider can ascertain the progress of the patient’s health. The proposed solution requires a constant ciphertext size and an element that can be stored on the blockchain to prove the authorship of data encryption. The existing multi-receiver signcryption does not appear relevant to our scenario. Therefore, the proposed multi-receiver signcryption has a constant ciphertext size with a small size of elements that can be stored on the blockchain to determine the sequence and authorship of the ciphertext.

Table 1 presents a summary of related work and identified research gaps, while Table 2 compares state-of-the-art research papers in digital twin technology applied to healthcare, categorized by focus, utility, paradigm, security, privacy, and framework. This comprehensive and well-organized overview of research papers serves as a valuable reference for understanding the various areas of focus and approaches in the digital twin field applied to healthcare.

**Table 1:** Comparison of reviewed literature with proposed solution.

Reference	Limitations	Our work
[15]	Does not specify model for data sharing	Provides one-to-one/many model for data sharing
	Does not provide any experimental results	Provides experimental results on operations/costs
	Unclear explanation of blockchain implementation.	Provides clear implementation of blockchain and operations
	Stores raw data off-chain	Stores only encrypted health records
	Seeks to virtualize healthcare as a service	Provides the digital twin to access services and data.
[29]	Work is not applicable to healthcare	Presents a digital twin that is healthcare-inclined
	No data confidentiality, a key point of health data sharing	Preserves confidentiality & integrity required in health data sharing
	No description of security parameters for storage of files.	Adequately describes the security for stored files
[30]	Uses symmetric encryption with no support for one-to-many sharing	Uses mIBSC encryption which supports one-to-many sharing
	Does not provide integrity controls for off-chain data storage	Uses inherent blockchain integrity controls
[31]	Focused on securing health data sharing between individual patients	Provides a digital twin that supports multiple user access
	Uses anonymity as a key security metric.	Requires identity for digital twin creation and usage
[24]	Does not protect data integrity, essential for secure data transactions	Guarantees data integrity, scheme thrives on signcryption & blockchain.
	Not applicable to health data sharing as there is no system model	Provides a system model, digital twin can access data/services.
	Protocol also lack verification.	Verification of protocol
[26]	Ciphertext sizes grow linearly with increasing number of receivers	Maintains static ciphertext size irrespective of no. of receivers
		Reduces communication and computation costs.
	Not applicable to data sharing as there is no system model	Provides system model, digital twin can securely access data
[28]	Ciphertext sizes grow linearly with increasing number of receivers	Maintains static ciphertext size irrespective of size of no. of receivers
		Reduces communication and computation costs.
	Not applicable to digital twin operations such as data contracts and service contracts.	Is based on digital twin operations.
	No sequential ordering for tracking digital twin authorship	Provides digital twin authorship tracking to check patient progress and completeness of patient data.

**Table 2:** A comparison of some state-of-the-art Digital Twin research papers in healthcare.

	[11]	[12]	[13]	[14]	[15]
**Focus**	Mo	IMP	Integration	Public health management	Structured data aggregation
**Utility**	SI	SI	Strategic care planning	Pandemic control (Covid-19)	Secure health data storage
**Paradigm**	PM	PC	Multi-agent systems	Smart City health	Multi-agent systems
**Security**	NP	NP	NP	NP	AP
**Privacy**	NP	NP	NP	NP	AP
**Framework**	NP	NP	Agent-based	Blockchain-based	Blockchain-based

AP = Applicable, NP = Non-applicable, Mo = Monitoring, PM = Precision medicine, Imp = Implementation, SI = Simulation, PC = Preventive care

## 3 Preliminaries

This section presents Digital Twins in healthcare, noting their properties. It also emphasizes the Blockchain, Smart Contracts and cryptographic notions.

### 3.1 Multi-receiver Identity-Based Signcryption

A multi-receiver identity-based encryption scheme (mIBSC) comprises four algorithms: Setup, Extract, Signcrypt, and Designcrypt, which are described as follows:

1. Setup(λ, *N*) → (*params*, *MSK*): The Setup algorithm takes a security parameter λ and *N* maximal size of the set of receivers for one encryption as input and provides a master secret key *MSK* and a set of public parameters *params* as the outputs.
2.
$\text{Extract}\left(MSK,ID_{i}\right)\to SK_{ID_{i}}$ : The Extract algorithm takes a master secret key, *MSK* and an identity, *ID*_*i*_ as input and provides the secret key $SK_{ID_{i}}$ as an output.
3.
$\text{Signcrypt}(m,S,ID_{Sender},SK_{ID_{Sender}})\to \sigma$ . The signcrypt algorithm takes in message *m*, the sender’s identity *ID*_*Sender*_ and the sender’s secret key $SK_{ID_{Sender}}$ and a set of identities of recipients *S* = {*ID*_1_, …, *ID*_*t*_}, with *s* ≤ *N*, and returns signcryption *σ* of *m* from $SK_{ID_{Sender}}$. The broadcast message to users in *S* is made up of (*S*, *ID*_*Sender*_, *C*_*T*_).
4.
$\text{Designcrypt}(\sigma ,S,ID_{Sender},ID_{i},SK_{ID_{i}})\to m$ : The Designcrypt algorithm takes in a signcryption *σ*, a subset *S* = {*ID*_1_, …, *ID*_*t*_}, with *s* ≤ *N*, the sender’s identity *ID*_*Sender*_, a receiver’s identity *ID*_*i*_ and the associated private key $SK_{ID_{i}}$. If *ID*_*i*_ ∈ *S*, the algorithm returns the message *m*. Otherwise it returns an error symbol ⊥.

Note that the public parameters *params* have been omitted for a concise description of the algorithms in mIBSC scheme. Due to page limitations the confidentiality and unforgeability security models of mIBSC have been omitted. Readers can refer to [25] for the security models.

### 3.2 Digital Twins

A patient Digital Twin is an evolving data-driven model that presents increasingly detailed approximations of a patient’s condition(s). Data from patients’ Electronic Medical Records (EMRs) and other relevant data sources can be combined and analyzed to produce a computational model to represent the patient digitally. Thus, analyses of the Digital Twin can facilitate predictions in sensitive areas such as experimental drug interactions [32], performance of tasks, create working models of organs, and study the behavior of physiological systems. The patient’s Digital Twin needs properties that facilitate analytics and other services. We describe these properties briefly below:

- **Adaptability**: For a patient Digital Twin, we define adaptability as the capacity to accept and incorporate changes to the Digital Twin so that it can adjust to suit predicted, anticipated, or stochastic changes. The virtual model must accommodate the changes that occur over its lifetime. Critically, the adaptability property provides the basis for other properties as well.
- **Extensibility**: The Digital Twin must be extensible to account for new parameters to be tracked for optimal operation and performance. For a patient’s Digital Twin, this is important for the several cycles of health conditions a patient may have over time. Extensibility allows the addition of new modules to enable functionality and capabilities so the Digital Twin can grow.
- **Modularity**: Aspects of the person that require monitoring can be virtualized and updated to provide insights for improved care. The patient Digital Twin can therefore be composed of several distinct but interconnected modules grouped into logical categories. These modules may represent physiological systems, conditions, etc. The modules can include all data relevant to patient care.
- **Connectivity**: Connectivity distinguishes the Digital Twin from other analytic models. Hence, we define connectivity as the capacity to connect to systems, platforms, and services to provide data for operating the physical asset. It may be updated periodically from data sources with changes in predefined categories of data, contexts, and conditions. The availability of new data, bandwidth, etc., can determine the frequency of connectivity.
- **Programmability**: The patient Digital Twin can support experiments by taking data inputs that can be processed to determine desirable outcomes within constrained boundaries and under specific conditions to support optimal health. Thus, a Digital Twin instance can test and adaptively refine treatment until the desired outcome is optimal. Conversely, failure of such experiments has no disadvantage for the patient since the Digital Twin can have multiple instances.
- **Flags**: The Digital Twin can represent a set of distinct conditions and system states that a physical system manifests under specified constraints. The Digital Twin can provide data to adaptively and preemptively manage the patient’s condition(s) through simulations. Thus, the twin can receive configurations that respond to each medical condition’s thresholds.

### 3.3 Blockchain

**Fig 2:**
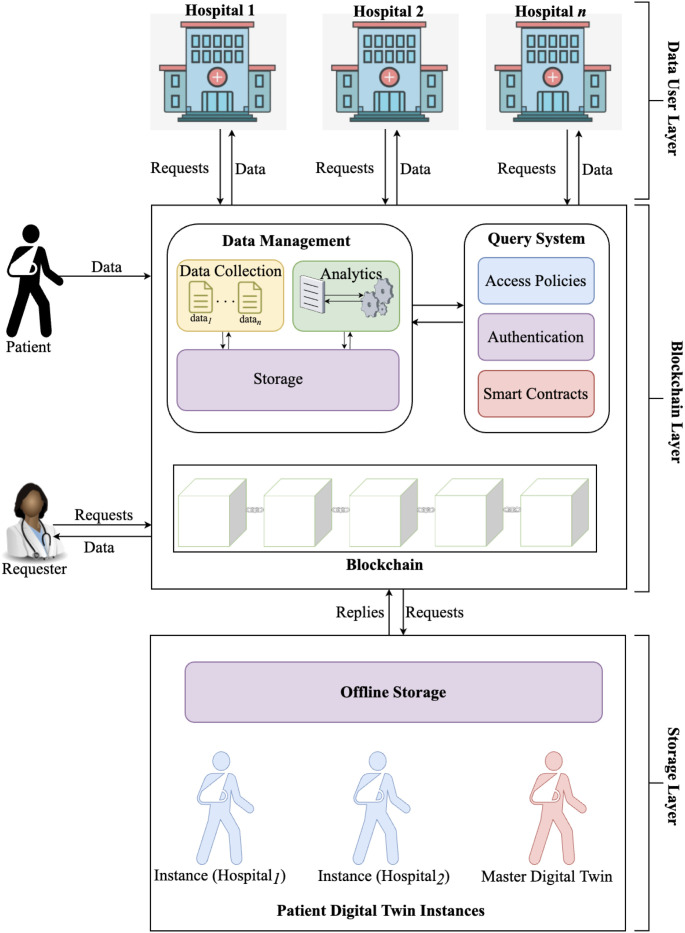
A visual representation of transactions on the blockchain.

The blockchain is a linked-list data structure and a consensus protocol initially designed to prevent double-spending in Bitcoin. Each block *b* in the blockchain *β* is a container that holds transaction data. The blockchain maintains an extending list of transactions such that each node contains copies of transactions accepted by the network. Transactions in the blockchain are immutable and pseudonymous, and users may generate a new address for each transaction to achieve credible anonymity on the network. Nodes collaborate to confer transaction validity through a consensus mechanism, so that a single coherent version of history is maintained as the basis for further action. This blockchain property provides behavioral data for developing the patient’s Digital Twin. Fig 2 depicts a visual representation of the blockchain showing how transactions are linked. We base our system on the blockchain to account for the following:

- **Preemptive Assumptions on User Behavior**: The patient Digital Twin is a data-sharing agent that provides insights on specific aspects of the patient’s health. A requester may exhibit behavior that deviates from care requirements, so capturing all requests/responses is crucial to account for connections between healthcare goals and outcomes. Thus, we capture instances of the patient Digital Twin for offline storage while proof of their existence is stored on the blockchain. The timestamps of Digital Twin instances and blockchain data are essential in preemptively constructing a timeline for the sequence of actions that definitively establish cause and effect.
- **Consistent View of Transactions**: For specialized care, patients’ mobility among several hospitals makes healthcare a collaborative venture. However, hospitals do not update one another on patients’ progress for obvious regulatory, competitive, and economic reasons. Thus, while a complete, consistent view of a patient’s medical history is beneficial, it may be unavailable. Using a patient Digital Twin instance for each hospital, the patient can maintain a master Digital Twin that synchronizes with the other instances after validation.
- **Immutability of Records**: Since a complete medical history is critical to the treatment, the blockchain can be used to create and sustain a distributed, immutable ledger of Digital Twin instances for the patient. This guarantees access to health records for caregivers in multiple institutions while ensuring that appending data to patient Digital Twin instances cannot be performed without the proper permissions. Thus, timestamped updates to Digital Twin instances and their hashes combine to provide greater security.

### 3.4 Smart contracts

**Fig 3:**
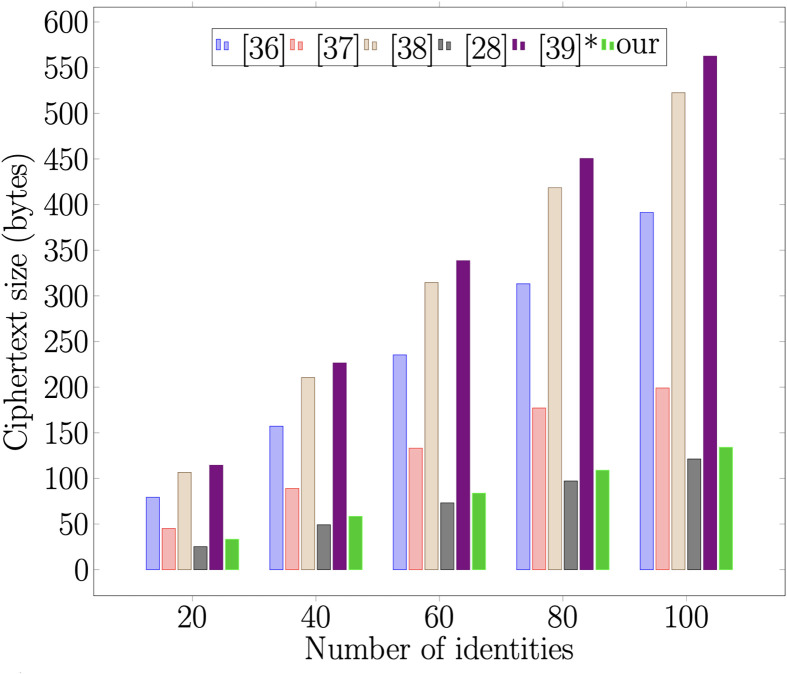
A conceptual overview of the Digital Twin as a construct of smart contracts.

We include the blockchain in our research to fully take advantage of the Smart Contract functionality. A *smart contract* is a script stored and executed on the blockchain by a connected node after meeting specific contract conditions. By encoding desirable actions as respondent scripts without specifying which node can perform them, we can ensure required interactions are censorship-resistant. It is critical to automate the predictable aspects of Digital Twin operations like updates and limit manual interactions by users other than the patient and approved caregivers. Smart contracts securely decentralize the Digital Twin update process by conceptualizing the patient as a set of interacting scripts, as shown in Fig 3. While smart contracts are not the only way to secure updates to the Digital Twin, they rely on other blockchain properties to offer extra layers of security through data provenance [33]. In this research, we used the Ethereum blockchain because of its global user base and support for smart contracts.

## 4 System overview

**Fig 4:**
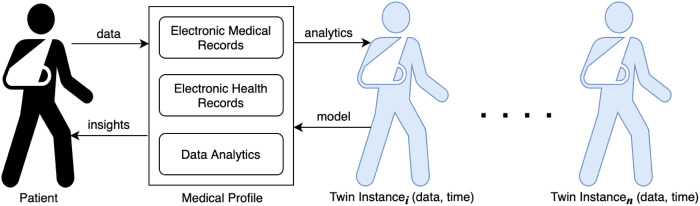
Overall system architecture for patient Digital Twin.

**Fig 5:**
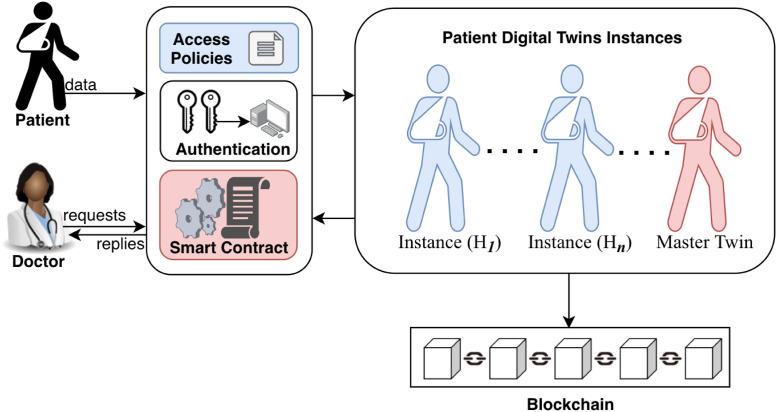
A visual representation of patient Digital Twin instances on the blockchain.

This section presents an overview of the proposed blockchain-secure patient Digital Twin system using smart contracts. Fig 4 shows the overall system architecture. The proposed system has several entities, including the data owner (patient), data users (hospitals), and the system itself, which includes data management, query system, and blockchain network. These entities work together to ensure seamless interactions and secure data sharing among the components of the system. Below is a brief description of the system entities and their functions:

1. **Data owner**:The patient is the owner of the data obtained from sensors, hospital records, and other health records required to create their digital twin. However, the franchise of the data is given to the hospital to maintain accurate health records to secure the wellness of the patient without any security breaches. Therefore, the hospital is the custodian of the data.
2. **Hospitals**: Hospitals play a crucial role in creating complete medical information for the patient to generate master records, which are accurate, fresh, computed, and sound to create a digital twin for the patient. Nurses and doctors who deal directly with the patient’s digital twin for good health provision are considered trustworthy to execute their roles without being an adversary for data breaches. The system ensures that encrypted data used in creating the patient digital twin is accessible only to those authorized in the hospital to avoid sensitive information falling into the wrong hands.
3. **Patient**: In the context of this research, a patient is defined as a human entity who seeks healthcare services from a hospital and is a primary data contributor in the healthcare system. The healthcare services that the patient receives require data sharing transactions with other entities within the hospital or outside of it. Hence, the patient’s medical record is essential for proper care and can also be shared with other healthcare providers as necessary upon authorised request.
4. **Query System**: The Query System has three components and processes users’ requests for access to the patient Digital Twin. The first is the Authentication module which verifies the source and destination of requests before they can be processed. It connects to an Access Policies module which is the second component to check for specific permissions patients define when they first register in the system as users. The third is the Smart Contracts module which executes the transaction of data access after the first two modules have successfully processed a user’s request.
5. **Data Management**: This component presents an interface for participating hospitals to provide data on patients. It receives patient data from the hospitals and assigns it to the respective Digital Twin after performing preliminary analytics to check for new content for updating the patient Digital Twin. It has three modules: Data Collection, Analytics, and Storage modules. The Storage is partitioned into two distinct areas of administration: online Storage for operational data and offline Storage for data at rest, such as inactive Digital Twin instances.
6. **Blockchain**: The blockchain network receives and stores completed transactions from network nodes. The data from complete transactions is first prepared into discrete blocks containing transaction details of import to patient care. In this study, we define the blockchain as a decentralized and distributed network of participating hospitals that collectively maintain a tamper-proof and transparent record of longitudinal patient data using consensus mechanisms, cryptographic algorithms, and smart contracts. The patient’s latest transaction hash is included in the current block, along with records of queries, access requests, and hashes of patient Digital Twin instances as shown in Fig 5. The blockchain network’s version of transactions takes precedence over any single institution’s records, ensuring transparency and accountability.

## 5 System design

In this section, we will present a thorough description of the proposed scheme’s design and provide an illustrative scenario of its application.

### 5.1 Multi-receiver Identity-Based Signcryption(mIBSC)

#### 5.1.1 *Setup*(λ, *N*) → (*params*, *MSK*)

The scheme’s security parameter is λ, and the maximum size of the collection of receivers is *N*. $G_{1},G_{2}$ are two prime groups of order *p* such that |*p*| = λ. $g\in G_{1}$ and $h\in G_{2}$ such that a bilinear map $e:G_{1}\times G_{2}\to G_{T}$. Lets call the number of bits required to indicate an identity and a message *n*_0_ and *n*_1_, respectively. Three hash functions are used: $H_{1}:{\left\{0,1\right\}}^{n_{0}}\to Z_{p}^{*},H_{2}:{\left\{0,1\right\}}^{n_{1}}\times G_{2}\to Z_{p}^{*}$, and $H_{3}:G_{2}\to {\left\{0,1\right\}}^{\left(n_{1}\right)+\left|G_{2}\right|}$. The PKG selects $\gamma \overset{\$}{\leftarrow }Z_{p}^{*}$ and calculates *w* = *g*^*γ*^ and *u* = *e*(*g*, *h*). The public parameters are as follows:

$$\begin{array}{cc} & params\leftarrow (w,u,h,h^{\gamma },\ldots ,h^{\gamma^{N}}) \end{array}$$

The Master Secret Key is

$$\begin{array}{c} MSK=(g,\gamma ). \end{array}$$

#### 5.1.2 $\text{Extract}\left(ID_{i},MSK\right)\to SK_{ID_{i}}$

The PKG runs the Extract algorithm with the input of the user identity *ID*_*i*_ and master secret key *MSK* = (*g*, *γ*). Upon successful validation of the *ID*_*i*_, the PKG computes the secret key as $SK_{ID_{i}}=g^{\left(\frac{1}{H_{1}\left(ID_{i}\right)+\gamma }\right)}$. As a patient medical data is tailored into a Digital Twin to predict how a patient would respond to a given medication, the health models and data must be stored chronologically. Hence, a doctor may be confident that the patient digital twin holds accurate data and that all computational results on the patient digital twin are correct. This permits the doctor to see how a patient digital twin responds to a set of data input over time. Before outsourcing medical data to a cloud server, the hospital employs a smart contract to establish blockchain proof to achieve immutable sequential order.

#### 5.1.3 Signcryption

Suppose a hospital with an identity *ID*_*Sender*_, and a private key $SK_{sender}=g^{\left(\frac{1}{H_{1}\left(ID_{Sender}\right)+\gamma }\right)}$ wants to signcrypt a patient’s health data and a Digital Twin which are denoted here as *m* such that *t* healthcare providers of the identities *ID*_1_, …, *ID*_*t*_ can access the data, it performs the following:

- Select $k\overset{\$}{\leftarrow }Z_{p}^{*},\leftarrow {\left\{0,1\right\}}^{n}$.
- Compute the following:
  - *C*_1_ = *w*^−*k*^
  -
$C_{2}=h^{k\cdot \prod_{i=1}^{S}\left(\gamma +H_{1}\left(ID_{i}\right)\right)}$

  - *C*_3_ = *m* ⋅ *u*^*k*^.
  -
$f=H_{2}\left(m,C_{1},C_{2},C_{3}\right)$

  -
$v=SK_{sender}^{-kf}$

- Output $\text{Signcryption}\left(m,S,ID_{Sender},SK_{ID_{Sender}}\right)$ of *m* as $\sigma =(C_{1},C_{2},C_{3},v,L)$, where L is the list of the recipients who can be authorized to designcrypt *σ*.

**Fig 6:**
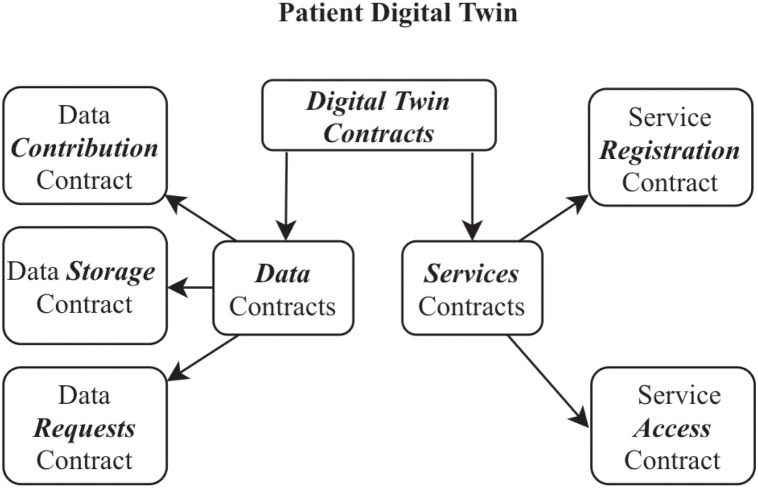
A visual representation of patient Digital Twin data request and response.

Here, we provide the details on how a patient digital twin is created. First, in Algorithm 1, a smart contract is deployed by the private key generator. It has to authorize a hospital before it uses Algorithm 2 to create an instance of the patient digital twin. Patients provide data to hospital data management platforms, as shown in the overall system architecture in Fig 4. To create the digital twin, one first provides a list of data sources that can later be updated. Ideally, these are hospital databases that host detailed patient data and online storage platforms for Personal Health Records from wearable sensors and other devices. A patient may have a digital twin for each unique condition such as disease progression, an organ, the whole body, etc. Thus, for each patient, doctors can access multiple digital twin instances, as shown in Fig 6. Formally, a digital twin instance *T*_*i*_, as proposed in this research, is a tuple of data sources, *σ* = [*σ*_1_, *σ*_2_, …, *σ*_*n*_], and identity of hospital *ID*_*H*_, patient *ID*_*P*_ and ciphertext auxiliary *v* which are encoded into smart contract as *v* = [*v*_1_, *v*_2_, …, *v*_*n*_] in Algorithm 2. The smart contract execution generates a cryptographic hash of the twin instance, *h*_*i*_, Merkel root, *mk*_*i*_, block number, *b*_*i*_, and a timestamp, *t*_*s*_, to facilitate proper sequencing of the digital twin instances. The digital twin instance *T*_*i*_ is represented as shown below.

$$\begin{array}{c} T_{i}=\left\{\sigma_{i},h_{i},mk_{i},b_{i},t_{s}\right\}. \end{array}$$ (1)

After being generated, the patient digital twin can be updated continuously to provide the details required for effective care. Hence, for this research, the primary smart contracts included, such as Algorithms 1 and 2, facilitate patient digital twin updates, access to health services, and provenance of the patient digital twin data. Finally, the hospital sends the digital twin instance *T*_*i*_ to the cloud server for sequential tracking of the various healthcare centers the patient visits. The EmitNotification alerts the hospitals about the new record update and alerts the hospital about the current digital twin instance.

**Algorithm 1**: AH: Authorized Hospitals

**Data**: Identity of hospital (*ID*_*H*_), Address (Addr)

**Result**: True/False

**1 struct** {

**2**  _*ID*_*H*,*A*_ *ddr*;

**3** } *T*;

**4** mapping(*ID*_*H*_ ⇒ *T*) AuthorizedHospitalList;

**5** AuthorizedHospitalList[*ID*_*H*_] ⋅_*ID*_*H*_ ← True;

**6** AuthorizedHospitalList[*ID*_*H*_]⋅_*Addr* ← True;

**7** EmitNotification(*ID*_*H*_,*Addr*, msg.sender) /* The EmitNotification trigger event on the blockchain which hospital *ID*_*p*_ can listen to get informed     */

#### 5.1.4 $\text{Unsigncryption}\left(S,\sigma ,ID_{sender},SK_{ID_{i}},ID_{i},params\right)$

To recover the message *m* from the ciphertext *σ*, a data user with the private key $SK_{ID_{i}}=g^{\left(\frac{1}{H_{1}\left(ID_{i}\right)+\gamma }\right)}$ and identity *ID*_*i*_ (*ID*_*i*_ ∈ *S*) performs the following:

1. Compute

$R={\left(e\left(C_{1},h^{p_{i,S}\left(\gamma \right)}\right)\cdot e\left(SK_{ID_{i}},C_{2}\right)\right)}^{\frac{1}{\prod_{j=1,j\neq 1}^{S}H_{1}ID_{j}}}$ with $p_{i,S}\left(\gamma \right)=\frac{1}{\gamma }\cdot \left(\prod_{j=1,j\neq i}^{S}\left(\gamma +H_{1}\left(ID_{j}\right)\right)-\prod_{j=1,j\neq i}^{S}H_{1}\left(ID_{j}\right)\right)$.
2. Recover the message as *m* = *C*_3_/*R* and compute $f=H_{2}\left(m,C_{1},C_{2},C_{3}\right)$
3. Accept the message *m* if $e\left(v,h^{\gamma }h^{H_{1}\left(ID_{sender}\right)}\right)\cdot R^{f}==1$; otherwise output the error symbol ⊥.

### 5.2 Correctness

Considering *σ* is well formed ciphertext for *S*:

$$\begin{array}{cc} R^{\prime } & =e\left(C_{1},h^{p_{i,S}\left(\gamma \right)}\right)\cdot e\left(SK_{ID_{i}},C_{2}\right)\\ & =e(g^{-k\cdot \gamma },h^{p_{i,S}\left(\gamma \right)})\cdot e(g^{\frac{1}{H_{1}\left(ID_{i}\right)+\gamma }},h^{k\cdot \prod_{j=1}^{S}\left(\gamma +H\left(ID_{j}\right)\right)})\\ & =e{\left(g,h\right)}^{-k\cdot (\prod_{j=1,j\neq i}^{S}\left(\gamma +H_{1}\left(ID_{j}\right)\right)-\prod_{j=1,j\neq i}^{S}H_{1}\left(ID_{j}\right))}\cdot \\ & e{\left(g,h\right)}^{k\cdot \prod_{j=1,j\neq i}^{s}\left(\gamma +H\left(ID_{j}\right)\right)}\\ & =e{\left(g,h\right)}^{k\cdot \prod_{j=1,j\neq i}^{S}H_{1}\left(ID_{j}\right)}\\ & =R^{\prod_{j=1,j\neq i}^{S}H\left(ID_{j}\right)}\\ & \text{Thus}\;R^{\prime \frac{1}{prod_{j=1,j\neq i}^{S}H_{1}\left(ID_{j}\right)}}=R=e{\left(g,h\right)}^{k} \end{array}$$

Then, the correctness of signature is performed as:

$$\begin{array}{cc} & e\left(v,h^{\gamma }h^{H_{1}\left(ID_{sender}\right)}\right)\cdot R^{f}==1\\ & e(g^{(\frac{1}{H_{1}\left(sender\right)+\gamma })-kf},h^{\gamma +H_{1}\left(ID_{sender}\right)})\cdot e{\left(g,h\right)}^{kf}==1\\ & {\left(g,h\right)}^{(\frac{H_{1}\left(ID_{sender}\right)+\gamma }{H_{1}(ID_{sender}+\gamma })-kf}\cdot e{\left(g,h\right)}^{kf}==1\\ & e{\left(g,h\right)}^{-kf+kf}==1 \end{array}$$

After successful unsigncryption of the ciphertext which is part of the digital twin data *T*_*i*_ recovered from the cloud server, the hospital, decryptor uses the block number *b*_*i*_ to confirm that the twin instance *h*_*i*_ the Merkel root *mk*_*i*_ and all other details of the digital twin are true on the blockchain. Note that the Merkel root guarantees the sequence of the digital twin.

**Algorithm 2**: PDTC: Patient Digital Twin Creation

**Data**: ciphertext auxiliary *v*, identity of hospital *ID*_*H*_, patient *ID*_*P*_

**1 struct** {

**2**  _*ID*_*H*_, _*ID*_*P*_, _*v*;

**3** } *T*;

**4** mapping(address ⇒ *T*) DataTwin;

**5** AH ah = AH();

 /* Algorithm 1 is called here      */

**6** **if** *ah⋅ AuthorizedHospitalList[ID_H_] and ah⋅ AuthorizedAddressList[msg.sender]* **then**

**7**  DataTwin[msg.sender]⋅_*ID*_*H*_ ← *ID*_*H*_;

**8**  DataTwin[msg.sender]⋅_*ID*_*P*_ ← *ID*_*P*_;

**9**  DataTwin[msg.sender]⋅_*v* ← *v*;

**10**  EmitNotification(*ID*_*H*_,*ID*_*s*_,*P*, msg.sender)

**11 end**

 /* The EmitNotification alerts the hospitals about the new record update */

### 5.3 Application scenarios

The proposed scheme for Blockchain-secure Patient Digital Twin in Healthcare using Smart Contracts can be applied in various domains of healthcare, such as chronic disease management, mental health disorders, patient monitoring, and personalized healthcare. The scheme can help improve the management of these health conditions by securely storing and accessing patient data on the blockchain, while also ensuring data confidentiality, integrity, and privacy through smart contract-based access control and cryptographic techniques.

One of the potential application of the proposed scheme is in managing mental health disorders. A patient with a mental health disorder can use wearable devices to collect data on their mood, sleep patterns, medication adherence, and other health metrics. The data can be securely stored on the cloud repository using Multi-receiver Identity-Based Signcryption (mIBSC) cryptographic technique for data confidentiality and integrity. The data is hashed on the blockchain for secure offline storage and protection of sensitive health information from unauthorized access.

The patient’s digital twin (virtual model that captures the essence of a patient’s medical conditions and interventions, based on the data collected from various sources, such as electronic medical records (EMR) and personal health records (PHR) from wearable devices) would contain a timestamped list of their medical conditions and corrective interventions, including information on medications, treatments, and therapy sessions. The patient digital twin could be used to monitor the patient’s progress over time, identify trends and patterns in their health data, and provide personalized recommendations for managing their mental health disorder.

For example, if the patient’s mood is consistently low at certain times of day, the digital twin could suggest adjustments to their medication regimen or therapy sessions. If the patient’s sleep patterns change, the digital twin could track the impact on their mood and adjust recommendations accordingly. Smart contracts can provide access control to the patient digital twin, enabling the patient to choose who can view and update their health information. The system could also monitor medication adherence and identify potential adverse drug interactions.

In summary, the proposed scheme can be a powerful tool for improving the management of healthcare and delivering personalized, data-driven care to patients. The use of mIBSC and smart contract-based access control would help ensure the privacy, security, and integrity of the patient’s health data, while also enabling secure offline data storage.

## 6 Security proof

Using the Gap Diffie-Hellman Exponent (GDDHE) assumption of [34], we demonstrate the IND-sID-CPA security of our system. We begin by defining the intermediate decisional problem as follows.

**Definition 1** *((f,g,F)-GDDHE). Let* $B=(p,G_{1},G_{2},G_{T},e\left(,\right))$ *denotes a bilinear map group system and let f and g represent two coprime polynomials with distinct pairwise roots with respective orders t and n. Let g*_0_ *denotes a generator of* $G_{1}$ *and h*_0_ *be a generator of* $G_{2}$ . *Solving the (f,g,F)-GDDHE problem consists*:

$$\begin{array}{cc} & g_{0},g_{0}^{\gamma },\ldots ,g_{0}^{\gamma^{t-1}},g_{0}^{\gamma \cdot f(\gamma )},g_{0}^{k\cdot \gamma \cdot f(\gamma )},\\ & h_{0},h_{0}^{\gamma },\ldots ,h_{0}^{\gamma^{2n}},h_{0}^{k\cdot g(\gamma )}, \end{array}$$

*The adversary* A *decides whether T* ∈ *e*(*g*_0_, *h*_0_)^*k*⋅*f*(*γ*)^ *or T is a random element in* $G_{T}$.

**Definition 2** *(l-SDHP Problem) The l-Strong Diffie– Hellman problem (l—SDHP) in the group G consists of, given* $g_{0},g_{0}\gamma ,\ldots ,g_{0}^{\gamma l}$ , *finding a pair* $\left(c,g_{0}^{\frac{1}{c+\gamma }}\right)$ *with* $c\in Z_{p}^{*}$ [18]

We denote by $Adv_{A}^{l-SDHP}$ the advantage of A in solving the (l—SDHP) in G and set $Adv_{A}^{l-SDHP}=Pr[A\left(g_{0},g_{0}\gamma ,\ldots ,g_{0}^{\gamma l}\right)=(c,g_{0}^{\frac{1}{c+\gamma }})]$, where $l,c\in Z_{p}^{*}$. The l—SDHP assumption is that, for any probabilistic polynomial time algorithm A, the advantage $Adv_{A}^{l-SDHP}$ is negligible.

### 6.1 Confidentiality

Let $Adv^{gddhe}\left(f,g,F,A\right)$ denotes the advantage of A in distinguishing the distributions (i.e., $T\in_{R}G$ or *T* ∈ (*g*_0_, *h*_0_)^*k*⋅*f*(*γ*)^, where ∈_*R*_ denotes random selection of an element in G).

**Corollary 0.1** *For any probabilistic algorithm* A *that sends at most q queries to the oracle, the adversary* A *has*:

$$\begin{array}{c} Adv^{gddhe}\left(f,g,F\right)\leq \frac{{\left(q+2\left(n+t+4\right)+2\right)}^{2}\cdot d}{2p} \end{array}$$

*where, d* = 2 ⋅ *max*(*n*, *t* + 1), *t* ∈_*R*_ *Z*_*q*_, *and n is the total number of identities*.

**Theorem 1** *For any n, t we have* $Adv_{mIBSC}^{\text{IND-sID-CPA}}\leq 2\cdot Adv^{gddhe}\left(f,g,F\right)$

Algorithm C is provided with the input $B=(p,G_{1},G_{2},G_{T},e\left(,\right))$, and a (f,g,F)-GDDHE instance in B (as described in Definition 1). Hence, we have f and g two coprime polynomials with pairwise distinct roots, of respective orders t and n, and C is given

$$\begin{array}{cc} & g_{0},g_{0}^{\gamma },\ldots ,g_{0}^{\gamma^{t-1}},g_{0}^{\gamma \cdot f(\gamma )},g_{0}^{k\cdot \gamma \cdot f(\gamma )},\\ & h_{0},h_{0}^{\gamma },\ldots ,h_{0}^{\gamma^{2n}},h_{0}^{k\cdot g(\gamma )}, \end{array}$$

and $T\in G_{T}$, decides whether *T* ∈ *e*(*g*_0_, *h*_0_)^*k*⋅*f*(*γ*)^ or *T* is a random element in $G_{T}$. We indicate that *f* and *g* are unitary polynomials for clarity, but this is not a requirement.

### 6.2 Notations

-
$f\left(X\right)=\prod_{i=1}^{t}\left(X+x_{i}\right),g\left(X\right)=\prod_{i=t+1}^{t+n}\left(X+x_{i}\right)$

-
$f_{i}\left(x\right)=\frac{f(x)}{x+x_{i}}$ for *i* ∈ [1, *t*], which is a polynomial of degree *t* − 1
-
$g_{i}\left(x\right)=\frac{g(x)}{x+x_{i}}$ for *i* ∈ [*t* + 1, *t* + *n*], which is a polynomial of degree *n* − 1

1. **Init**: The adversary A commits a set $S^{*}=ID_{1}^{*},\ldots ,ID_{t^{*}}^{*}$ of identities that it wants to attack (with t* ≤ *n*).
2. **Setup**: To produce the system parameters, C sets $g=g_{0}^{f(y)}$ (i.e. without computing it) and sets
  $$\begin{array}{cc} h= & h_{0}^{\prod_{i=t+S^{*}+i}^{t+n}\left(\gamma +x_{i}\right)},w=g_{0}^{\gamma \cdot f\left(\gamma \right)=g^{\gamma }},\\ v & =e{\left(g_{0},h_{0}\right)}^{f\left(\gamma \right)\cdot \prod_{t+s^{*}+1}^{t+n}\left(\gamma +x_{i}\right)}=e\left(g,h\right). \end{array}$$
  Eventually, C defines the public parameters as $params=(w,u,h,h^{\gamma },\ldots ,h^{y^{N}})$. Note that the challenger C is restricted from accessing the element *g*. C runs A on the system parameters $B,H_{1},H_{2}$ and params. Here, the hash oracles $H_{1},H_{2}$ are controlled by C.
3. **Query Phase 1**: At any point in time, the adversary A can query the following random oracles. To answer the queries, C maintains two lists $L_{H_{1}}$ and $L_{H_{2}}$.
  -
$H_{1}$ queries: The list $L_{H_{1}}$ contains at the beginning:
    $$\begin{array}{c} {\left\{\left(*,x_{i},*\right)\right\}}_{i=1}^{t},{\left\{\left(ID_{i},x_{i},*\right)\right\}}_{i=t+1}^{t+s^{*}} \end{array}$$
    (We select * to represent an empty element in $L_{H_{1}}$. When A decides to query on identity *ID*_*i*_,
    (a) If *ID*_*i*_ already exists in the list $L_{H_{1}}$, C answers with *x*_*i*_.
    (b) Else, C sets $H_{1}\left(ID_{i}\right)=x_{i}$ and completes the list with (*ID*_*i*_, *x*_*i*_, *).
  -
$H_{2}$ queries: To respond to this query, C keeps a list of tuples known as $L_{H_{2}}$ list. Each entry in this tuple is of the form (*m*, *C*_1_, *C*_2_, *C*_3_). At the beginning of the list, it is empty. To respond to queries, algorithm C performs the following:
    - If the queries on (*m*, *C*_1_, *C*_2_, *C*_3_) is in the list (*m*, *C*_1_, *C*_2_, *C*_3_, *f*), then respond with $f=H_{2}\left(m,C_{1},C_{2},C_{3}\right)$.
    - Else, C selects a random $f\in Z_{p}^{*}$ and updates $L_{H_{2}}$ list with (*m*, *C*_1_, *C*_2_, *C*_3_, *f*). C outputs *f* to A.
4. **Extraction queries**: The challenger C runs $O_{Extract}$ on *ID*_*i*_ ∉ S* and sends the associated private key $SK_{ID_{i}}$ to the adversary A. To generate the keys,
  - If A has already issued an extraction query on *ID*_*i*_, C responds with the associated $SK_{ID_{i}}$ in the list $L_{H_{1}}$.
  - Otherwise, if A already issued a hash query on *ID*_*i*_, then C uses the associated *x*_*i*_ to generate $SK_{ID_{i}}=g_{0}^{f_{i}\left(\gamma \right)}=g^{\frac{1}{\gamma +H_{1}\left(ID_{i}\right)}}$ and then updates the list $L_{H_{1}}$ with $SK_{ID_{i}}$ for *ID*_*i*_.
  - Otherwise, C sets $H_{1}\left(ID_{i}\right)=x_{i}$, generates the associated $SK_{ID_{i}}$ exactly as stated earlier and completes the list $L_{H_{1}}$ with $SK_{ID_{i}}$ for *ID*_*i*_.
5. **Challenge** At some point in time, C decides that phase 1 is over, challenger C computes $\text{Signcrypt}\left(m,S^{*},ID_{Sender},SK_{ID_{Sender}},params\right)\to \sigma^{*}$, where
  $$\begin{array}{cc} C_{1}= & g_{0}^{-k\cdot \gamma \cdot f(\gamma )},C_{2}=h_{0}^{k\cdot g(\gamma )},C_{3}=m_{b}\cdot T^{\prod_{i=t+s^{*}+1}^{t+n}x_{i}}\cdot \\ & e(g_{0}^{k\cdot \gamma \cdot f(\gamma )},h_{0}^{q^{\gamma }})\\ f= & H_{2}\left(m_{b},C_{1},C_{2},C_{3}\right),v^{*}=SK_{sender}^{-kf} \end{array}$$
  Here the challenger C randomly selects *b* ← 0, 1 and sets *m* = *m*_*b*_. C returns $\sigma^{*}=\left(C_{1},C_{2},C_{3}^{*},v^{*}\right)$. with $q\left(\gamma \right)=\frac{1}{\gamma }(\prod_{i=t+s^{*}+1}^{t+n}\left(\gamma +x_{i}\right)-\prod_{i=t+s^{*}+1}^{t+n}\left(x_{i}\right))$. One can verify that
  $$\begin{array}{cc} C_{1} & =w^{-k},\\ C_{2} & =h_{0}^{k\cdot \prod_{i=t+s^{*}=1}^{t+1}\left(\gamma +x_{i}\right)\cdot \prod_{i=t+1}^{t+s^{*}}\left(\gamma +x_{i}\right)}\\ & =h^{k\cdot \prod_{i=t+1}^{t+s^{*}}\left(\gamma +H_{1}\left(ID_{i}^{*}\right)\right)} \end{array}$$
  Note that if *T* = *e*(*g*_0_, *h*_0_)^*k*⋅*f*(*y*)^, then *C*_3_ = *m*_*b*_ ⋅ *u*^*k*^.
6. **Query Phase 2**: This phase is same as phase 1. The adversary A continues to issue queries with the restriction that no extraction query is committed on *ID*_*i*_ ∈ S*.
7. **Guess**: Finally, A returns a guess *b*′ ∈ {0, 1} and wins the game if *b* = *b*′. One has
  $$\begin{array}{cc} & Adv^{gddhe}\left(f,g,F,C\right)\\ & =Pr\left[b^{\prime }=b|real\right]-Pr\left[b^{\prime }=b|rand\right]\\ & =\frac{1}{2}\times (Pr\left[b^{\prime }=1|b=1\wedge real\right]-\\ & Pr[b^{\prime }=1|b=0\wedge real])\\ & =\frac{1}{2}\times (Pr\left[b^{\prime }=1|b=1\wedge rand\right]-\\ & Pr[b^{\prime }=1|b=0\wedge rand]) \end{array}$$
  In the random situation, the distribution of *b* is independent of the adversary’s point of view. *Pr*[*b*′ = 1|*b* = 1 ∧ *rand*] − *Pr*[*b*′ = 1|*b* = 0 ∧ *rand*]. All simulations are perfect, the distributions of all variables defined by C absolutely conform with the semantic security game. Therefore $Adv_{mIBSC}^{\text{IND-sID-CPA}}\left(t,nA\right)=Pr\left[b^{\prime }=1|b=1\wedge real\right]-Pr\left[b^{\prime }=1|b=0\wedge real\right]$. Putting it together, yield $Adv^{gddhe}\left(f,g,F,C\right)=\frac{1}{2}\cdot Adv_{mIBSC}^{\text{IND-sID-CPA}}\left(t,nA\right)$.

### 6.3 Unforgeability

Assume that EUF-CMA adversary A making *l* extraction queries, qHi queries to random oracles *Hi*(*i* = 1, 2) and *q*_*s*_*c* signcryption queries, has an advantage $e\leq 10\left(q_{sc}+1\right)\left(q_{sc}+q_{H_{2}}\right)/2k$ against the proposed scheme. Then, there is an algorithm *R* to solve the (*l* + *N*)–*SDHP* with advantage

$$\begin{array}{c} e^{\prime }\geq \frac{1}{9}. \end{array}$$

*R* provides the input $\left(h,h^{\gamma },\ldots ,h^{\gamma^{l+N}}\right)$ and aims to find a pair $\left(c,h^{\frac{1}{c+\gamma }}\right)$. In a setup phase, it constructs a generator $G\in G_{1}$ such that it knows *l* − 1 pairs $\left(x_{i},G^{\frac{1}{x_{i}+\gamma }}\right)$ for $x_{1},\ldots ,x_{l-1}\overset{\$}{\leftarrow }Z_{p}^{*}$. The challenger C performs the following:

- Select $\eta \overset{\$}{\leftarrow }Z_{p}^{*}$ and set *P* = *h*^*η*^.
- Select $x_{1},\ldots ,x_{l-1}\overset{\$}{\leftarrow }Z_{p}^{*}$ such that $f\left(z\right)=\prod_{i=1}^{l-1}\left(z+x_{i}\right)$ to get $c_{0},c_{1},\ldots ,c_{l-1}\overset{\$}{\leftarrow }Z_{p}^{*}$ with $f\left(z\right)=\sum_{i=0}^{l-1}c_{1}z^{i}$.
- Set elements $H=h^{\sum_{i=0}^{l-1}c_{i}\gamma^{i}=h^{f(\gamma )}}$ and *G* = *H*^*η*^ = *p*^*f*(*γ*)^.
- Compute $h^{\sum_{i=1}^{l}c_{l-1}\gamma^{i}}=H^{\gamma },H^{\gamma^{2}},\ldots ,H^{\gamma^{N}}$ and make $\langle G^{\gamma },H^{\gamma },H^{\gamma^{2}},\ldots ,H^{\gamma^{N}},e\left(G,H\right)\rangle$ public.
- Let 1 ≤ *i* ≤ *l* − 1 and expand $f_{i}\left(z\right)=\frac{f(z)}{\left(z+x_{i}\right)}=\sum_{i=0}^{l-2}d_{i}z^{i}$ and $p^{f_{i}\left(\gamma \right)}=G^{\frac{1}{x_{i}+\gamma }}$.

A gives C the target user identity $ID_{\ell }^{*}$ on which A wants to forge a signature. C then prepares to respond to A’s queries throughout the game. It starts by setting the counter *i* to 1. We will assume that $H_{1}$ queries are distinct for the sake of simplicity, and that any query involving an identity *ID*_*i*_ is preceded by the random oracle query $H_{1}\left(ID_{i}\right)$.

-
$H_{1}$ **queries**: On the input of the identity *ID*_*i*_ by A, C returns a random $x_{\ell }\overset{\$}{\leftarrow }Z_{p}^{*}$ if $ID_{i}=ID_{\ell }^{*}$. Else, C responds *x*_*i*_ and increases *i*. C stores (*ID*_*i*_, *x*_*i*_) in a list $L_{H_{1}}$. Note $H_{2}$ query is same as in the confidentiality proof, so it is omitted here.
- **Key generation queries on** $ID_{i}\neq ID_{\ell }^{*}$ : C retrieves the matching pair (*ID*_*i*_, *x*_*i*_) from $L_{H_{1}}$ and outputs the previously computed $G^{\frac{1}{\gamma +x_{i}}}$ Note: No extraction query on $ID_{\ell }^{*}$ can be executed.
- **Forgery**: Signcryption query on $\left(m,ID_{A},ID_{1},ID2,\ldots ,ID_{n}\right)$: If $ID_{A}\neq ID_{\ell }^{*}$, proceeds normally as in the Signcrypt algorithm. Otherwise, C performs the following:
  - Select $k\overset{\$}{\leftarrow }Z_{p}^{*}$.
  - Compute the following:
    -
$C_{1}^{*}=w^{(\gamma +x_{A})k}$

    -
$C_{2}^{*}=H^{\left(\gamma +x_{A}\right)k\cdot \prod_{i=1}^{n}\left(x_{i}+\gamma \right)}$

    -
$C_{3}^{*}=m\cdot u^{k}$ .
    -
$f^{*}=H_{2}\left(m,C_{1},C_{2},C_{3}\right)$

    -
$v^{*}=G^{\frac{1}{(\gamma +x_{A})-kf}}$

  - Add the elements $\left(m,C_{1}^{*},C_{2}^{*},C_{3}^{*},f^{*}\right)$ to $L_{H_{2}}$ list.
  - Output signcryption of *m* as $\langle C_{1}^{*},C_{2}^{*},C_{3}^{*},v^{*},L\rangle$, where L is the list of recipients who are authorized to designcrypt σ*.
- **Unsigncryption** $\left(S,\sigma^{*},ID_{\ell }^{*},SK_{ID_{i}},ID_{i},params\right)$ : C looks up $L_{H_{2}}$ for an entry of the form $\left(m,C_{1}^{*},C_{2}^{*},C_{3}^{*},f^{*}\right)$ and checks whether it satisfies the following condition:
  $$\begin{array}{cc} & e\left(G^{\frac{1}{(\gamma +x_{A})-kf}},H^{\gamma }H^{H_{1}\left(ID_{\ell }^{*}\right)}\right)\cdot {\left(G,H\right)}^{kf}\overset{?}{=}1\\ & e{\left(G,H\right)}^{(\frac{x_{A}+\gamma }{H_{1}\left(ID_{\ell }^{*}\right)+\gamma })-kf}\cdot e{\left(G,H\right)}^{kf}\overset{?}{=}1 \end{array}$$
  The case in which A can generate a valid ciphertext is by correctly guessing the hash value $x_{A}=H_{1}\left(ID_{\ell }^{*}\right)$ without querying on $\left(ID_{\ell }^{*}\right)$. However, this event occurs only with a negligible probability of $\frac{1}{2^{\lambda }}$.
  Note that, with the forking lemma, A does not perform key generation queries on $ID_{i}\neq ID_{\ell }^{*}$. Based on the theory of irreflexivity, *R* can generate the message-signature from σ* with the private key $sk_{ID_{\ell }}$. Since identity-less chosen message attack is possible with a forking lemma [35], we unify the sender’s message *m* and identity $A_{\ell }$ as a fake message $\left(A_{\ell },m\right)$. Supposing A is an effective forger, then there exists a very powerful algorithm $A^{\prime }$ which can produce a pair of signed messages $\left(\left(A_{\ell },f^{*},k^{*}\right),v^{*}\right)$ and $\left(\left(A_{\ell },f,k\right),v\right)$, where *f* ≠ f* under the same commitment. $\hat{C}$ interacts with $A^{\prime }$and A to solve the ECDL problem as follows:
  1. Based on the forking lemma in [35], by executing A’, $\hat{C}$ can derive two distinct equations from the signatures ((*ID*_*ℓ*_, *m*, *f*, *k*), *v*) and $\left(\left(ID_{\ell }^{*},m,f^{*},k^{*}\right),v^{*}\right)$ as:
    $$\begin{array}{c} e\left(G^{\frac{1}{(\gamma +x_{A_{\ell }})-kf}},H^{\gamma }H^{H_{1}\left(ID_{\ell }^{*}\right)}\right)\cdot {\left(G,H\right)}^{kf}=1 \end{array}$$ (2)
    $$\begin{array}{c} e\left(G^{\frac{1}{\left(\gamma +x_{A_{\ell }}\right)-k^{*}f^{*}}},H^{\gamma }H^{H_{1}\left(ID_{\ell }^{*}\right)}\right)\cdot {\left(G,H\right)}^{k^{*}f^{*}}=1 \end{array}$$ (3)
  2. Since both Eqs 2 and 3 satisfy the relations:
    $$e\left(G^{\frac{1}{(\gamma +x_{A_{\ell }})-kf}},H^{\gamma }H^{H_{1}\left(ID_{\ell }^{*}\right)}\right)\cdot {\left(G,H\right)}^{kf}$$ (4)
    $$=e\left(G^{\frac{1}{\left(\gamma +x_{A_{\ell }}\right)-k^{*}f^{*}}},H^{\gamma }H^{H_{1}\left(ID_{\ell }^{*}\right)}\right)\cdot {\left(G,H\right)}^{k^{*}f^{*}}$$ (5)
  Then, Set $T^{*}=v/v^{*}=G^{\frac{1}{(\gamma +x_{A_{\ell }})-kf}}/G^{\frac{1}{\left(\gamma +x_{A_{\ell }}\right)-k^{*}f^{*}}}=G^{\frac{1}{ID_{\ell }^{*}+\gamma }}$ (Here, $x_{A_{\ell }}=H_{1}\left(ID_{\ell }^{*}\right)$). From T*, R first obtains *a*_−1_, … *a*_*l*−2_ for which $\frac{f(z)}{\left(z+x_{A_{\ell }}\right)}=\frac{a-1}{\left(z+x_{A_{\ell }}\right)}+\sum_{i=0}^{l-2}a_{i}z^{i}$ and computes
  $$\begin{array}{c} v^{*}={\left[T^{*}\cdot P^{\prod_{i=0}^{l-2}a_{i}\gamma_{i}}\right]}^{\frac{1}{a_{1}}}=P^{\frac{1}{x_{\ell }+\gamma }} \end{array}$$
  and $\eta^{-1}\cdot v^{*}=h^{\frac{1}{x_{\ell }+\gamma }}=h^{\frac{1}{H_{1}\left(ID_{\ell }\right)+\gamma }}$ since *P* = *h*^*η*^. Eventually, R outputs the $\left(x_{\ell },h^{\frac{1}{x_{\ell }+\gamma }}\right)$ as the solution to (l + N)—SDHP.
  As in [22], if $Adv_{A}^{\text{mIBBSC}}\geq 10\left(q_{sc}+1\right)\left(q_{sc}+q_{H_{2}}\right)/2^{k}$, where *l* extraction queries, $q_{H_{i}}$ queries to random oracles *H*_*i*_(*i* = 1, 2) and *q*_*sc*_ signcryption queries are made, then $Adv_{R}^{(l+N)R–SDHP}\geq 1/9$.

## 7 Efficiency evaluation

This section evaluates the efficiency of our scheme as it relates to computational and storage overheads, and the deployment of smart contracts.

### 7.1 Computational overhead

**Fig 7:**
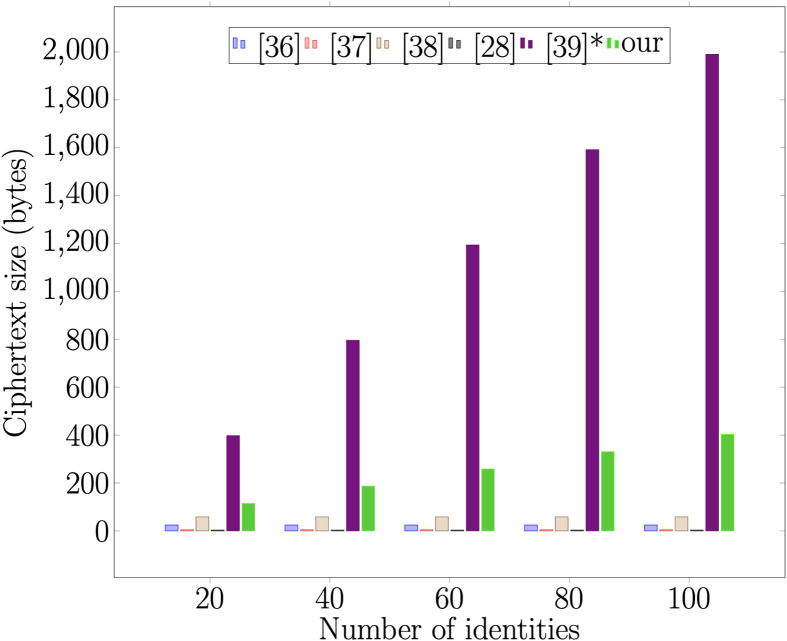
Signcryption.

**Fig 8:**
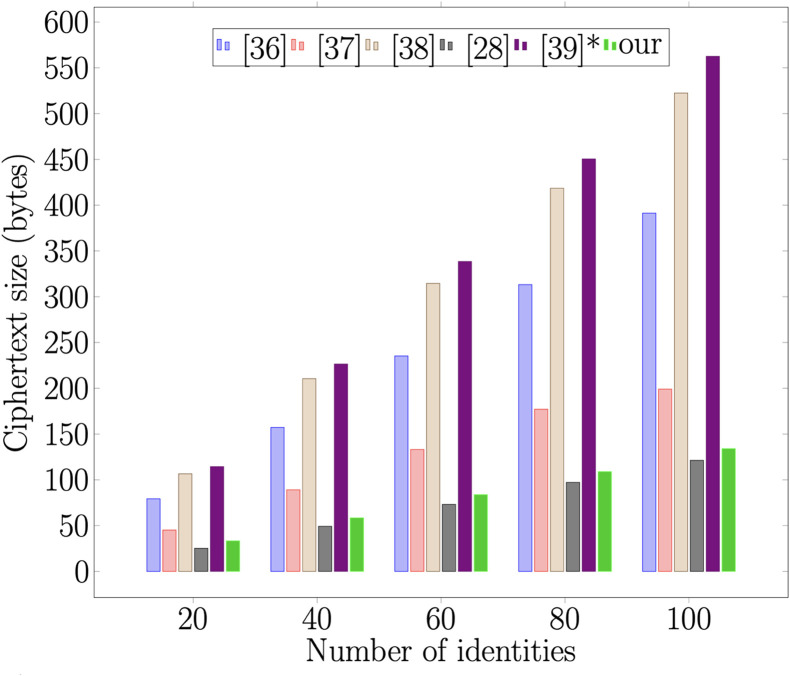
Unsigncryption cost.

To demonstrate the efficiency of the proposed scheme relative to other schemes, we perform computation analysis with recent broadcast signcryption schemes: [28, 36–39]. The experiment was conducted on a Windows desktop computer with a 2.0GHz Intel Core i7 processor and 8GB 1600 MHz DDR3 RAM. We used Multi-Precision Integer and Rational Arithmetic C Library (MIRACL), a C++ cryptographic library. The execution times are based on the average of 300 trials. The results of the execution are shown in Table 3. In Table 3, we define the related symbols to indicate the computational complexity of the operations. However, only the operations in the table are considered in this paper. Other operations, such as addition, subtraction, and hashing, with little or insignificant computational time, are ignored. The theoretical comparison of our proposed scheme and other related works is shown in Table 4. The computation cost benchmarks are shown in Figs 7 and 8. Although the proposed scheme has a high computation cost compared to other related schemes, when we consider pre-computation of the element, the cost of signcryption becomes $3\bar{E}$ while unsigncryption becomes $3\bar{P}$. Hence, the proposed scheme is efficient in communication and computational aspects. The pre-computation becomes applicable when the set of receivers remains the same as in the previous session. Both broadcasters and receivers can reuse the previous information. The difference is significant because the computation operations are reduced to $3\bar{P}$ for unsigncryption on the client side.

**Table 3:** Running times of time-consuming operations.

Operation	Timing (ms)
Elliptic curve group exponentiation ($\bar{E}$)	1.26
Bilinear pairing($\bar{P)}$	14.32
Pairing-based scalar point multiplication (*M*_1_)	4.34
Elliptic curve point multiplication (*M*_2_)	0.98

**Table 4:** Comparison of computational cost and communication cost.

Scheme	Signcryption	Unsigncryption	Ciphertext size
[36]	$\left(3n+1\right)\bar{E}$	$M_{1}+3\bar{P}+\bar{E}$	$3|G_{1}\left|+n\right|Z_{p}^{*}\left|+|ID\right|$
[37]	(2*n* + 1)*M*_2_	4*M*_2_	$2|G_{1}\left|+\left(S+2\right)\right|Z_{p}^{*}|$
[38]	$\left(4n+2\right)\bar{E}$	$\bar{E}+4\bar{P}$	$\left(2n+3\right)|G_{1}|$
[28]	(*n* + 1)*M*_2_	3*M*_2_	$2|G_{1}\left|+\left(n+2\right)\right|Z_{p}^{*}|$
[39]*	$nM_{1}+\left(n+5\right)\bar{E}$	$nM_{1}+\left(n+2\right)\bar{E}+3\bar{P}$	$3|G_{1}\left|+\left(n+1\right)|ID\right|$
Ours	$M_{1}+\left(3+n\right)\bar{E}$	$3\bar{P}+nM_{1}+n\bar{E}$	$4|G_{1}\left|+\right|Z_{p}^{*}|$

[39]* = Proposal I

### 7.2 Communication overhead

**Fig 9:**
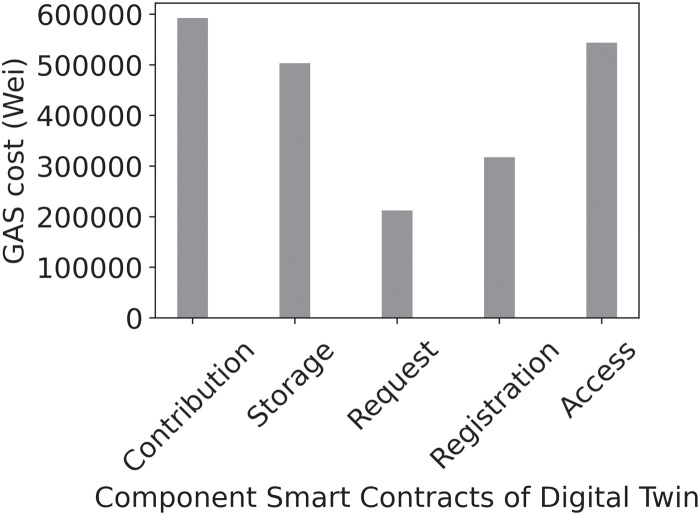
Ciphertext size.

Additionally, we examine the communication overhead of the proposed scheme and other related schemes. As in [40], we undertake the size of elements $|G_{1}|=1024$ bits, $|G_{2}|=1024$ bits and |*m*| = 160 bits. The five schemes are compared in Table 4. The benchmark result in Fig 9 demonstrates unequivocally that the proposed scheme achieves the objectives. The prime objective of the proposed scheme is to have the smallest ciphertext size so that the element which is stored on the blockchain will not increase when the attribute size increases.

### 7.3 Discussion of smart contract deployment and evaluations

We assume the presence of a sensor that can transmit continuous physiological data from the patient to the aggregation platform represented in our research by the Data Management module. Patients’ increasing use of wearable sensors validates our assumption in this respect. The Data Management module collects and processes data using the analytics component to sort through received signals to distinguish status data (such as positioning) from care data, such as inputs made by doctors and other caregivers. The output from the analytics module is stored by the connected offline storage till the Query System receives requests for data, or smart contracts are executed to create a patient digital twin instance and to update the master digital twin. The metadata for the transaction, such as hashes, timestamps and commitments, are then prepared into a block and transmitted to the blockchain.

For the experiment, we used the Ropsten Test Environment to test several smart contracts grouped into two categories: Data Contracts and Service Contracts. The Data Contracts handle events relating to data contribution, storage and requests. The Service Contracts deal with services to patients. Each smart contract is invoked using its address on the test Blockchain. Each individual contract was developed using the solidity programming language with the Remix IDE. The appropriate amount of ether was provided by Infura at 4 ETH for all tests needed to run on an Ethereum Decentralized Node with more than 2000 test nodes providing an acceptable degree of consensus. The resources for this experiment were a minimum transaction fee of 0.0002 ETH and a gas rate of 0.27 US dollars per transfer. The computer on which the experiments were performed was an Ubuntu Linux desktop configured with a 1.5 TB Solid State Drive hard drive, 16 GB RAM, and an Intel Core i7 CPU running at 2.67 GHz.

**Fig 10:**
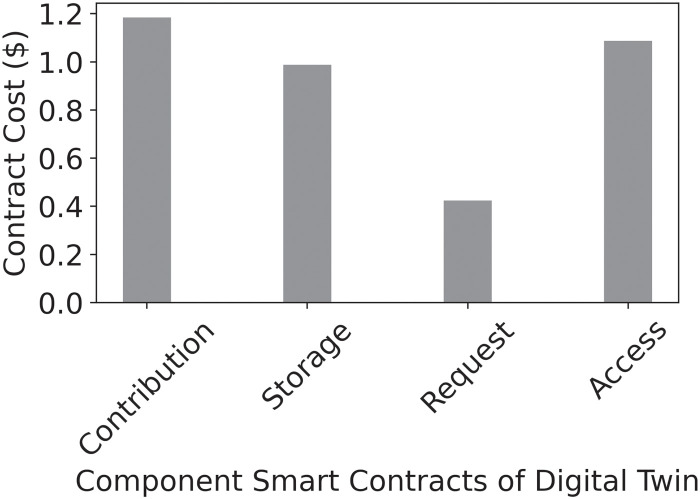
Smart contracts costs in wei.

**Fig 11:**
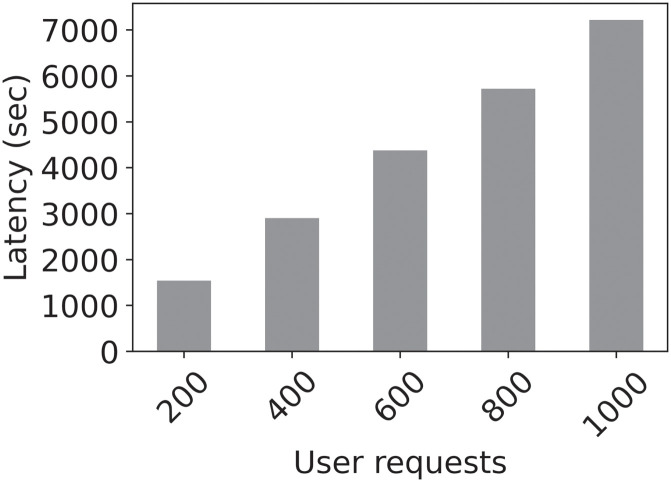
Smart contracts costs in US dollars.

**Fig 12:**
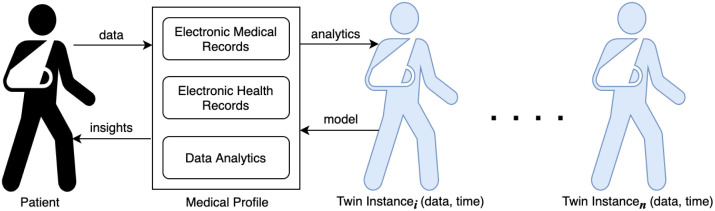
Latency per number of requests in the system.

Smart contracts-based patient digital twins can effectively and economically automate patient activities in healthcare considering the current high costs. For example, with the Data Contracts that manage data acquisition and sharing tasks, we measured a total deployment cost of 1308303 Wei on Ethereum, which amounts to about $2.6153, a competitive amount for accessing healthcare. Figs 10 and 11 present the costs of deploying smart contracts in dollars and in Wei while Fig 12 shows the cumulative latency for increasing numbers of user requests. Thus, the aggregate latency for 200 requests in the scheme is 1600 seconds, i.e., 8 seconds per request. The average block confirmation time was approximately 11.7 seconds. The low costs of transactions in both time and monetary terms coupled with our system’s provable security make it an effective tool for health data sharing. Even in the unlikely event of a dispute, the immutable records and timestamped transactions provide sufficient input for fault tracing and effective resolution.

Researchers have conducted studies on the use of blockchain in healthcare, focusing on secure data sharing. Most research emphasize the blockchain properties of immutable transactions and distributed storage. None have considered hosting a collection of smart contracts to act as a data-sharing agent on behalf of the patient, as proposed in this research. Hospitals’ data sharing requirement for patient care makes our proposed smart contracts-based patient digital twin a necessary addition to healthcare innovation. Thus, we compare our proposed system to blockchain-based health data-sharing papers, each of which has been cited more than 200 times. The comparison is made in Table 5.

**Table 5:** Comparison of our work with other frameworks for blockchain health data sharing.

Metrics	[36]	[37]	[38]	[28]	[39]	Ours
Blockchain-based	N	N	N	Y	N	Y
Digital Twin-based	N	N	N	N	N	Y
Access Control	Y	Y	Y	Y	Y	Y
Senders and Receivers Known	N	N	N	N	Y	Y
Data Privacy-Preserving	Y	Y	Y	Y	Y	Y

## 8 Conclusion

Modern healthcare places unprecedented focus on patient-centered care, which requires secure communication among multiple parties. The process depends on the secure sharing of patient data and can be tedious for those involved. Thus, automation of a data-sharing mechanism with agency such as the patient digital twin can promote efficient interaction between the entities required to administer patient care. This paper proposes a blockchain-secure patient digital twin as a secure construct for personal health data sharing. We use smart contracts on the Ethereum network to ensure that patients have control over their medical records with guaranteed privacy and security. We protect the data and instances of the digital twins generated using proven cryptographic techniques that are also computationally light. We evaluate our research with some experimental results and comparison with other works. Our proposed system can be integrated into existing healthcare platforms using a permissioned blockchain for maximum privacy and security. We hope to extend the research to provide the patient digital twin with greater autonomy.

## Supporting information

S1 File (ZIP)

S2 File (ZIP)
